# Condom Use and Prevalence of Genital *Chlamydia trachomatis* Among the Korean Female Sex Workers

**DOI:** 10.4178/epih/e2010008

**Published:** 2010-08-13

**Authors:** Joongyub Lee, Sun-Young Jung, Dong Seok Kwon, Minsoo Jung, Byung-Joo Park

**Affiliations:** 1 Medical Research Collaborating Center, Seoul National University Hospital, Seoul, Korea.; 2 Department of Preventive Medicine, Seoul National University College of Medicine, Seoul, Korea.; 3 Korea Federation for HIV/AIDS Prevention, Seoul, Korea.; 4 Department of Health Policy and Management, School of Public Health, Seoul National University, Seoul, Korea.

**Keywords:** *Chlamydia trachomatis*, Condom use, Sex worker

## Abstract

**OBJECTIVES:**

Since 2004, availability of resources for preventing sexually transmitted diseases in Korean female sex workers (FSWs) has decreased because of strict application of a law against prostitution. This study is to evaluate the condom use and prevalence of *Chlamydia trachomatis* among FSWs in Korea.

**METHODS:**

We performed a cross-sectional study of FSWs from 15 major sex work sites in Korea from June to November 2008, using convenience sampling. Self-administered questionnaires and urine samples were collected after all participants' written informed consent. Urine samples were analyzed with PCR at a single central laboratory.

**RESULTS:**

Among 1,086 FSWs who consented to study participation, data from 999 FSWs were appropriate for analysis. *C. trachomatis* prevalence was 12.8% (95% CI: 10.7-14.9%). Younger age increased risk for *C. trachomatis*. Whereas majority of FSWs (71.0%) answered high self confidence in condom negotiation, the proportion of FSWs who always used condoms last month was only 23.7%. However, practicing regular condom use showed significant protection against chlamydia infection, not self confidence in condom negotiation.

**CONCLUSION:**

In Korea, FSWs were not practicing enough self-protection at work with a high prevalence of *C. trachomatis*. Education for constant practice of protection against sexually transmitted diseases is needed, especially for younger FSWs.

## INTRODUCTION

*Chlamydia trachomatis* is the most common pathogen of human genitourinary system infection, annual number of new cases was estimated as 92 million [1]; a large proportion of the patients are asymptomatic and stay untreated. However, untreated *C. trachomatis* infection can cause chronic inflammation in the genital organs, which results in serious health problems like pelvic inflammatory disease (PID) and infertility [2].

It is not feasible to approach a study on female sex workers (FSWs) academically and produce reliable statistics, for that reason epidemiologic data are very limited regarding the prevalence of sexually transmitted diseases (STDs) among FSWs in developing countries, especially in those where the prostitution is illegal like Korea. In September 2004, the Act on the Prevention of Sexual Traffic and Protection, etc. of Victims Thereof became effective, and the police clamped down on traditional sex work venues. The budget for the healthcare of FSWs was cut, and it became more difficult for non-governmental organizations (NGOs) to provide services for FSWs. Therefore, individual FSW have been in decreased supply of condoms and contraceptives after 2004 which would induce an increased risk of STDs for FSWs. Thus we urgently needed to evaluate the current status of condom use and STD control among FSWs.

We performed a cross-sectional study to evaluate the condom use and *C. trachomatis* prevalence in FSWs.

## MATERIALS AND METHODS

We conducted a cross-sectional study from June to November 2008 with the collaboration of an NGO, the Korea Federation for HIV/AIDS Prevention. The NGO contacted every representative business owners of 39 sex work venues identified in a nationwide economic survey of the sex industry in 2003. Total 24 out of 39 representatives responded positively to our request to participate. We excluded 9 small venues considering efficiency, convenience of sampling by site. Sample size calculation was done assuming that the size of the target population would be 6,009, which was an estimation from the Korean Ministry of Gender Equality report of 2003. Assuming that estimate, the calculated sample size was 1,063 with a *C. trachomatis* prevalence of 30% and a precision of 2.5% [3]. Questionnaire was developed through cultural adaptation of previously validated questionnaires for sexual behavior including condom use [4].

After the informed consent, a self-administered questionnaire on sexual behavior, and random urine samples were obtained. On completion of the survey, a card was given in which the telephone number of an information center and a 10-digit individual code were printed to assure participants' access to their test results. We provided those who had positive results with a sincere recommendation for receiving prompt and adequate treatment.

After the manual review of questionnaires, data were input via double entry. Urine samples were analyzed at a central laboratory with a commercial PCR kit (BDProbTecET, BD Diagnostic Systems).

Condom use, general characteristics were presented with proportion of each characteristic, and prevalence of *C. trachomatis* was calculated with 95% confidence interval.

Univariate logistic regression was performed to evaluate the relationship between general characteristics, condom use and *C. trachomatis* prevalence. Variables with a p-value<0.2 were selected as covariates for the multiple logistic regression model to calculate the adjusted prevalence odds ratios (PORs) and 95% confidence intervals (CIs). SPSS version 17.0 (SPSS INc., Chicago, IL, USA) was used for analysis.

The Institutional Review Board of Seoul National University Hospital and the Seoul National University College of Medicine reviewed and approved the study protocol.

## RESULTS

We obtained written consent from 1,083 FSWs. However, 18 FSWs refused to provide questionnaires or samples during the survey. After exclusion of the 22 incomplete or low quality questionnaires and 44 inappropriate samples, we used the data from 999 FSW for statistical analysis.

We found that 128 samples were positive for *C. trachomatis* out of 999 samples; the proportion with a positive test was 12.8% (95% CI: 10.7-14.9%). Distribution of general characteristics was described in Table 1. Older age (p<0.01), smoking more than 1 pack/day (p=0.14), previous STD history (p=0.06), anxiety for STDs (p=0.10) and low educational level (p=0.18) were included in the multiple logistic regression model.

When asked about the frequency of condom use, only 23.7% of participants answered 100% condom use during last month, and 36.0% reported 100% use of condom on the day before the survey. However 71.0% of participants answered that they have high self confidence in condom negotiation (Table 2). The higher the inconsistency of the condom use, the higher the prevalence of *C. trachomatis* (p for trend<0.01). This tendency appeared in both condom use at last night and during last month, and remained significant after adjustment of potential confounding variables. However, increasing level of self confidence in condom negotiation did not show decreasing trend of *C. trachomatis* prevalence (Table 2).

## DISCUSSION

In this study results, not self confidence, but consistent condom use practice was strongly associated with low prevalence of *C. trachomatis*. Condom use has been shown to have protective effect against STDs including *C. trachomatis* [5, 6], which was reproduced in our study, but proportion of participant who always used condom was smaller than previous studies which had been conducted in Cambodia by Wong et al. [7] and in China by Pingmin et al. [8] Wong et al. interviewed 140 direct FSWs and reported that 78% of FSWs used condom always with client [7]. Pingmin et al. reported 58.8% of 'every time' condom use during 1 month period before the survey [8]. What we found during the literature review brought about a hypothesis that the minor difference in the questionnaire item for condom use frequency could bring about a difficulty in direct comparison of the result except for the 100% use. A survey of 7 major venues that a congress member's bill was based on reported that 60.5% out of 1,000 FSWs 'always' used condom at work in 2006 [9]. Although no other published Korean data from comparable population before the law was available, our study results suggested decreased prevalence of consistent condom use.

Several interpretations can be raised for increase of inconsistent condom use. Selection of low utilization group by the emigration is one of them; The sex industry in South Korea, like other Asian developing countries, had been reorienting itself from direct prostitution to indirect prostitution [10], and this change was anticipated to be accelerated by the law. Moreover, it was believed that this law had brought the so-called 'balloon effect' causing the change in structure and visibility of prostitution, and migration of FSWs to neighboring countries or indirect sex work via informal regional linkages in the sex industry. During this process if the most adaptive FSWs to this environment were selected, selection bias can be a possible explanation to our findings.

Confidence in condom negotiation was not related to the *C. trachomatis* prevalence. However, all the participants of this study were direct FSW. Even though some of participants have high condom negotiation skill, they were at high risk of *C. trachomatis* infection when they use condom less than 90% of coitus.

We included 5 variables (age, education, smoking, STD history, and anxiety for STD) as potential confounders, but only age was significant after the adjustment in the final model, which was consistent with previous reports that younger age was a risk factor for *C. trachomatis* infection [11]. Although the peak prevalent age range of STD (16-19 years old) was not covered in this study, linear trend for the risk increase along age was significant (p for trend<0.01).

A *C. trachomatis* prevalence of 12.8% (95% CI: 10.7-14.9%) seems higher than those observed in studies for the general population of Korea. The Korean Center for Disease Control and Prevention (KCDC) reported a 4.7% *C. trachomatis* prevalence in 959 females from 4 major cities in 2007 [12], and Lee et al. reported 3.1% in 420 female college students from the Seoul metropolitan area [13]. In addition, we could find studies with similar prevalence among FSW in Bangladesh and Cambodia [14, 15], while recent FSW studies conducted in Spain and San Francisco showed relatively smaller values in prevalence than our study [16, 17]. However, without standardization for the age structure of the population, a simple comparison may not be appropriate.

A large study population, which was obtained by covering most of the major sex work sites in Korea, is merit of this study. But limited availability of information on the size and distribution of FSWs made it impossible for us to perform probability sampling, which may put limitation on external validity. We found that FSWs were not practicing enough self-protection at work with a high prevalence of *C. trachomatis*. Education for constant practice of protection against STDs is needed, especially for younger FSWs.

## Figures and Tables

**Table 1:**
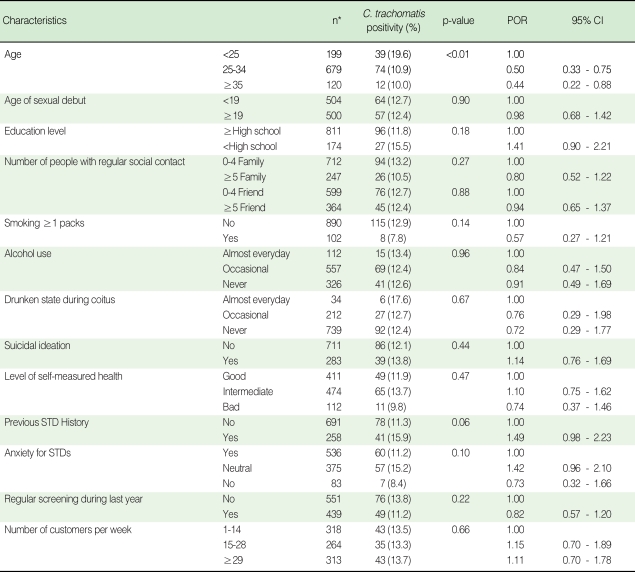
Characteristics of study participants and prevalence odds ratio (POR) of *C. trachomatis*

^*^Numbers may not add up because of missing values in each variable. STD, sexually transmitted disease; CI, confidence interval.

**Table 2:**
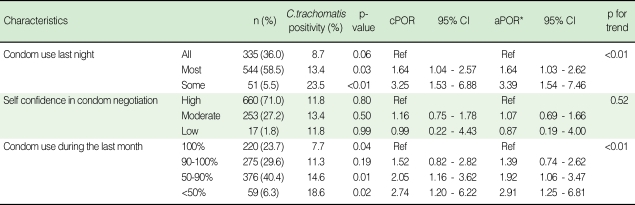
Characteristics of condom use and impact of condom use on the prevalence of *C. trachomatis* in 930 participants by adjusted prevalence odds ratio and p for trend

^*^Adjusted for age, education, previous sexually transmitted disease history, anxiety for the sexually transmitted disease, smoking more than 1 pack/day. cPOR, crude prevalence odds ratio; CI, confidence interval; aPOR, adjusted prevalence odds ratio.
